# Herbivory and Relative Growth Rates of *Pieris rapae* are Correlated with Host Constitutive Salicylic Acid and Flowering Time

**DOI:** 10.1007/s10886-015-0572-z

**Published:** 2015-04-17

**Authors:** Andrew Lariviere, Lisa B. Limeri, George A. Meindl, M. Brian Traw

**Affiliations:** Department of Biological Sciences, University of Pittsburgh, 4249 Fifth Ave, Pittsburgh, PA 15260 USA

**Keywords:** Life history, Defense, Crosstalk, Herbivory, Brassicaceae, Oviposition, *Pieris rapae*

## Abstract

Treatment of plants with exogenous salicylic acid (SA) improves resistance to many bacterial pathogens, but can suppress resistance to insect herbivores. While plants vary naturally in constitutive SA, whether such differences are predictive of resistance to insect herbivores has not been studied previously. We examined the possible role of this endogenous SA in structuring the interactions between the cabbage white butterfly, *Pieris rapae*, and ten hosts in the mustard family (Brassicaceae). Because *P. rapae* has multiple generations that utilize different hosts across the year, we included five spring-flowering mustards and five summer-flowering mustards that co-occur in ruderal habitats in upstate New York. Under common garden conditions, the spring flowering mustards (*Capsella bursa-pastoris, Draba verna, Cardamine impatiens, Barbarea vulgaris,* and *Arabidopsis thaliana*) were significantly more resistant to *P. rapae*, supporting 42 % less herbivory (*P* = 0.015) and 64 % lower relative growth rates (*P* = 0.007), relative to the summer flowering mustards (*Sisymbrium altissimum*, *Brassica nigra, Sinapis arvense, Lepidium campestre,* and *Arabis canadensis*). Leaf total constitutive SA explained significant variation in larval herbivory (*R*^*2*^ = 75.3 %, *P* = 0.007) and relative growth rates (*R*^*2*^ = 59.4 %, *P* = 0.043). The three species with the lowest levels of constitutive SA (*Capsella bursa-pastoris, Draba verna,* and *Cardamine impatiens*) were the most resistant to larvae. *Barbarea vulgaris* and *Arabis canadensis* were notable exceptions, exhibiting high SA concentrations and intermediate resistance to *P. rapae*. These results suggest a curvilinear relationship between leaf constitutive SA and the herbivory by *P. rapae,* and they provide some insight into the ecology and possible management of this economically important crop pest.

**Electronic supplementary material:**

The online version of this article (doi:10.1007/s10886-015-0572-z) contains supplementary material, which is available to authorized users.

## Introduction

Insect herbivores and pathogens cause significant reductions in the performance of plants in nature (Crawley 1983) and in agriculture (Oerke 2006). Plants, in turn, have evolved a sophisticated set of defensive responses that are mediated in large part by the hormones salicylic acid (SA) and jasmonic acid (Karban 2011). Much of what we know about these hormones has come from the study of a few major model systems (Vlot et al. 2009). Little is known about how concentrations of these hormones vary in natural communities of plants and the extent to which this variation may structure plant – enemy interactions in nature (but see Todesco et al. 2010; Zhang et al. 2014, 2015).

Application of exogenous SA or of plant activators that operate via the SA pathway decreases symptoms of disease in agricultural crops (Vallad and Goodman 2004). However, an interesting indirect effect of the application of exogenous SA to plants has been the induction of increased susceptibility to insect herbivores (Cipollini et al. 2004; Thaler et al. 1999, 2002a). This is an example of what is collectively referred to as ecological costs of defense, where the act of defending against one particular enemy makes a plant more susceptible to other enemies (Johnson et al. 2014; Karban 2011; Thaler et al. 2002a, b). In a meta-analysis of the literature, Strauss et al. (2002) found that 62 % of studies that investigated plant defense against insect herbivores reported an ecological cost of resistance, suggesting that such ecological costs may be widespread in plants.

While exogenous treatment of plants with SA has been shown to increase the susceptibility to insect herbivores, the effects of constitutive variation in SA on plant resistance to insect herbivores has not been reported previously. Constitutive tissue concentrations of SA do differ among genotypes within species (Silverman et al. 1995; Zhang et al. 2014) and among species (Raskin 1992). Constitutive differences in allocation to SA among genotypes from natural populations of the widely-studied mustard, *Arabidopsis thaliana*, also have been shown recently to influence resistance to bacterial and fungal pathogens (Todesco et al. 2010; Zhang et al. 2014) and to vary geographically across an environmental gradient (Zhang et al. 2015).

Constitutive SA concentrations in plants have been linked previously to plant phenology and the transition to flowering, specifically (Jin et al. 2008; Martinez et al. 2004; Wada et al. 2010). Plants that have their major growth and flowering stages during the spring, such as spring ephemeral mustards, are likely to experience different herbivore and pathogen pressures relative to plants that emerge and bloom in the summer (Feeny 1976, 1977). Some evidence has suggested that spring ephemeral mustards experience lower levels of herbivory from insect herbivores (Gaines and Kok 1995; Vail et al. 1991) and have lower levels of constitutive resistance against damage than do summer mustards (Feeny and Rosenberry 1982). The effects of plant life history on resistance to herbivores have been studied previously (Silvertown and Dodd 1996; Van Zandt 2007), but not in relation to basal SA concentrations.

Because neonate butterfly larvae, particularly of specialists, typically cannot switch hosts and therefore die if placed on an incorrect host, it is critical that adult females oviposit on acceptable hosts. Female butterflies are known to use leaf chemistry in selecting host plants (Dethier 1982; Renwick and Chew 1994) and have been shown to prefer host plants that promote larval growth and development (Chew 1977; Mayhew 1997). If constitutively high SA concentration suppresses production of constitutive jasmonic acid (JA)-mediated defenses, then it is possible that female butterflies may prefer plants with constitutively high SA concentrations, as these may provide the best food source for their progeny.

We focus here on a group of ten mustard species that co-occur spatially in marginal environments across the Northeastern United States, and all interact with Pierid butterflies (Gaines and Kok 1995; Renwick and Radke 1987; Slansky and Feeny 1977). This group is notable first because it includes the genetic model plant, *Arabidopsis thaliana*, and several other species (e.g., *Capsella bursa-pastoris*) for which full genome sequences are currently available. Likewise, *Pieris rapae* is one of the best studied butterflies and co-occurs with these mustards across much of the US and Europe (Capinera 2001). This system also is notable because these mustards, while all occurring in ruderal habitats (e.g., agricultural edges, railroad beds, stream washes, and trailsides) possess substantial variation in life history strategies. In the Northeastern US, five of the species (*A. thaliana*, *C. bursa-pastoris*, *Draba verna*, *Cardamine impatiens*, and *Barbarea vulgaris*) typically flower in March, April, and May, whereas the other five species (*Arabis canadensis*, *Brassica nigra*, *Lepidium campestre*, *Sinapis arvensis*, and *Sisymbrium altissimum*) flower in June, July, and August (Uva et al. 1997). In New York and Pennsylvania, this difference in flowering phenology has been consistent across years (B. Traw, pers. observation). Each of the ten species in this focal group co-occurs in close proximity with at least three other species in the group. As such, emerging *P. rapae* females typically have several of these mustard species to choose from within very close proximity to each other.

In this study, we addressed aspects of the hypothesis that constitutive SA production can affect herbivore resistance. We measured first whether leaf herbivory rates, larval relative growth rates, or oviposition rates by *P. rapae* females differed among the spring- and summer-flowering species under common garden conditions. We then asked whether the differences that we observed corresponded to the underlying leaf SA concentrations. Finally, we assessed whether host plant selection by the maternal butterflies correlates with larval performance on these same hosts.

## Methods and Materials

### Plant Material and Growth Conditions

Seeds of all ten mustards (*Arabis canadensis, Arabidopsis thaliana, Barbarea vulgaris, Brassica nigra, Capsella bursa-pastoris, Cardamine impatiens*, *Draba verna, Lepidium campestre*, *Sinapis arvensis*, *Sisymbrium altissimum*) were collected from ruderal sites in Tompkins County, NY in the vicinity of Cornell University. Seeds were pooled from at least twenty maternal plants collected from multiple populations where possible. Seeds were sown on Pro-Mix BX potting soil (Premier Tech, Quakertown, PA, USA) in 36-well flats and placed in a 4 °C cold-room for 3 d of cold stratification. Flats then were transferred to an environmentally-controlled growth chamber at the University of Pittsburgh with constant conditions of 22 °C, 12 h day-night cycle, and 350 μmol m^−2^ sec^−1^ light provided by a 1:1 mixture of sodium and metal halide lamps. All species were grown simultaneously, watered as needed, fertilized every 10 d with 10 ml of Peter’s 20:20:20 fertilizer (full strength), randomized, and moved at least once per week within the growth chamber to minimize positional effects.

### Larval Performance Assays

We tested larval performance on the 10 mustard species using first instars of *P. rapae* that were obtained from the laboratory of Dr. Nathan Morehouse at the University of Pittsburgh. We used first instars because they would not be potentially influenced by prior diet. We conducted the assays in sterile 12-well plates on Dec 12–14, 2012. Each plate contained leaf disks from all 10 mustard species, leaving two wells empty. Each well was assigned randomly to receive one leaf disk (8 mm diam) collected by hole punch from the largest leaf of a new plant of one of the 10 species. All leaf disks were used within 30 min of being taken. Plants and larvae were not reused. Filter paper moistened with distilled water was placed on the bottom of each well to prevent leaf disks from drying out. Each larva was weighed individually to the microgram on a MX5 Microbalance (Mettler Toledo) prior to being placed on the leaf disks. Lids were placed on the plates, and plates were maintained in a growth chamber with conditions as stated above for the plants. After 24 h, caterpillars were reweighed, and the area of disk consumed was estimated visually for each well by using a standard method (Utsumi et al. 2009). We conducted five replicate assays, each in a new sterile 12-well plate for all 10 species, for a total of 50 plants and larvae sampled (Table 1). One end of one plate was lost when the filter paper was not moistened, leaving four replicates for some of the species (*Draba verna*, *Cardamine impatiens*, *Arabidopsis thaliana*, *Sinapis arvensis*, and *Brassica nigra*). The relative growth rate (RGR) of the caterpillars was calculated as follows:

**Table 1:** Comparison of means (+/- 1SE) for spring vs. summer flowering mustards from the ruderal community in upstate New York

Flowering	Name	*N*	Percent herbivory (% disk eaten)	Relative growth rate (g ^*^ g^−1^_*_d^−1^)	*N*	Oviposition rate (eggs^*^f^−1^_*_d^−1^_*m_^−2^)	Free salicylic acid (μg^*^g^−1^ dry mass)	Total salicylic acid (μg^*^g^−1^ dry mass)
Spring	*Capsella bursa-pastoris*	5	1.0 +/− 1.0	−0.05 +/− 0.03	4	0.0 +/− 0.0	0.4 +/− 0.01	0.09 +/− 0.02
	*Draba verna*	4	45.0 +/− 21.6	0.05 +/− 0.06	4	12.7 +/− 9.3	0.03 +/− 0.01	0.17 +/− 0.05
	*Cardamine impatients*	4	55.0 +/− 20.6	0.25 +/− 0.07	4	1.5 +/− 0.9	0.08 +/− 0.02	0.18 +/− 0.06
	*Barbarea vulgaris*†	5	59.4 +/− 19.1	0.27 +/− 0.13	4	30.4 +/− 9.7	0.85 +/− 0.21	2.65 +/− 0.61
	*Arabidopsis thaliana*	4	78.7 +/− 12.6	0.44 +/− 0.13	4	29.8 +/− 11.4	0.12 +/− 0.01	0.25 +/− 0.03
Summer	*Sinapis arvensis*	4	78.7 +/− 18.0	0.63 +/− 0.10	4	128.8 +/− 50.0	0.17 +/− 0.03	0.29 +/− 0.07
	*Arabis canadensis*	5	79.0 +/− 18.6	0.53 +/− 0.11	4	3.4 +/− 2.1	1.55 +/− 0.13	1.59 +/− 0.19
	*Lepitium campestre*	5	95.0 +/− 5.0	0.43 +/− 0.06	4	1.5 +/− 1.2	0.53 +/− 0.11	0.72 +/− 0.16
	*Sisymbrium altissimum*	5	98.0 +/− 1.2	0.51 +/− 0.07	4	59. +/− 22.5	0.34 +/− 0.04	0.61 +/− 0.03
	*Brassica nigra*	4	98.7 +/− 1.2	0.54 +/− 0.14	4	147.1 +/− 32.4	0.37 +/− 0.05	0.87 +/− 0.13

†*Bararea vulgaris* had three replicates for free and total SA

$$ \mathrm{R}\mathrm{G}\mathrm{R}=\left( \ln \kern0.24em \left(\mathrm{finalweight}\right)- \ln \kern0.24em \left(\mathrm{initialweight}\right)\right)/\mathrm{day}. $$

### Female Oviposition Choice Test

To assess female oviposition preferences among the mustards, we conducted trials on 4-wk-old plants in which individuals of each of the 10 species were placed together in a mesh cage (0.5, 0.5, and 1 m for width, length, and height), which was then exposed to natural light. Gravid female *P. rapae* butterflies, mated within the previous 24 h, were obtained from the Morehouse lab at the University of Pittsburgh, and placed in the cage with the plants. The availability of females differed for each trial, and so the numbers of females used were 30, 15, 23, and 15 in Trials 1, 2, 3, and 4, respectively, which were conducted during February 14 – 25, 2013. The number of eggs deposited on each plant was counted at the end of the trial. Each trial consisted of a new set of 10 plants and naïve females, for a total of 40 plants and 83 naïve female butterflies (Table 1). Each trial was terminated when 5 % of the females had died, which occurred at 36, 60, 48, and 120 h in Trial 1, 2, 3, and 4, respectively. To determine the leaf area of each plant, we traced all leaves and digitized the images as described previously (Traw and Feeny 2008). Oviposition rate for each plant was calculated as the number of eggs laid there divided by the number of females, the length of the trial in days, and the amount of leaf area available.

### Measurement of Leaf SA Concentration

We measure leaf SA concentrations in a separate common garden experiment that included four replicate plants for each of the 10 mustard species, for a total of 40 plants. We harvested the above ground portion of each plant on November 21, 2011 when plants were 4-wk-old by cutting at the base with a razor blade and placing the rosette in a coin envelope and immediately submerging it in liquid nitrogen. We stored the tissue at −80 °C prior to assessment of leaf SA concentration. Samples then were transferred from the freezer to dry ice and immediately lyophilized for 3d to freeze-dry the tissue and then pulverized. Salicylic acid extraction followed a standard method (Dewdney et al. 2000). Approximately 20–25 mg of dry leaf tissue were weighed and suspended in 3 ml of 90 % methanol. As an internal run control, we added 1 μg of *o-*anisic acid (Sigma # 169978) to each sample (100 μl of a 10 μg/ml solution in 100 % methanol), and placed tubes in a shaker at 200 rpm at room temperature for 24 h. We transferred the liquid to a new tube, resuspended the pellet in 3 ml of 100 % methanol, and repeated the extraction. The supernatant fractions from the two extractions were combined and vortexed. Because SA exists in both free and sugar-conjugated forms in plants, we split each sample into equal volumes into two screwcap tubes. The first aliquot was used to measure free SA, while the second was used to measure total SA, which includes the portion conjugated to sugar. We first dried all tubes in a fume hood to remove the methanol, and then added 40U of β-glucosidase (Sigma # 0395) in 400 μl of 100 mM sodium acetate buffer (pH 5.5) to the first aliquot to liberate the SA from its glucoside. We added 400 μl of buffer to the other aliquot, but no enzyme. All samples were incubated overnight at 37 °C and then received 400 μl of 10 % trichloroacetic acid to end the reaction. To separate SA from more polar compounds, we partitioned all samples twice with 1 ml of an organic extraction solvent (100:99:1 of ethyl acetate: cyclopentane: 2-propanol), vortexing each time, and collecting the two organic phase fractions together in a centrifuge tube, which we then evaporated to dryness. We resuspended the samples in 600 μl of 55 % methanol, vortexed, and agitated them overnight. To remove any remaining impurities, we centrifuged the samples at 5000 g for 15 min, transferred the supernatant onto 0.2 μm nylon spin-prep membrane filters (Fisher #07-200-389), centrifuged at 14,000 g, and loaded the samples into small vials. To measure concentrations of SA by HPLC, we used an HP1100 (Agilent # G1380-90000) system with a 4.6 × 150 mm Eclipse XDB C-18 column (Agilent # 993967-902) and fluorescence detector (excitation at 301 nm and emission at 412 nm for SA and excitation at 301 nm and emission at 365 nm for *o*-anisic acid). Solvent flow was 1 ml/min, beginning with 30 % of 100 % methanol and 70 % of 0.5 % acetic acid for 5 min, increasing to 40 % methanol at 7.5 min, and 60 % methanol at 15 min, returning to 30 % methanol at 18 min. To calculate concentrations (μg/g leaf dry mass) of free and total SA, we divided the peak area of each compound by the product of the peak area of the *o*-anisic acid internal standard and sample mass.

### Statistical Analyses

Data were natural log transformed prior to analysis. To assess the difference between spring-flowering and summer-flowering species, we performed a nested ANOVA with the mustard species nested with flower time group for the following three variables: oviposition rate, free SA, and total SA, which all had balanced numbers of replicates per species. Two of the variables, percent herbivory and relative growth rate, had unbalanced numbers of replicates measured for each species. For those two variables, we calculated species averages from the available replicates, and we then assessed the difference between the five spring and five summer-flowering species by one-way ANOVA. Least squares linear regression was performed to assess the relationship between herbivore performance and leaf SA concentration. Polynomial regression was performed when the residuals from the linear regression exhibited a curvilinear relationship with the predictor variable. All calculations were performed using Minitab v. 17.1 (Minitab Inc., State College, PA, USA).

## Results

**Fig. 1:**
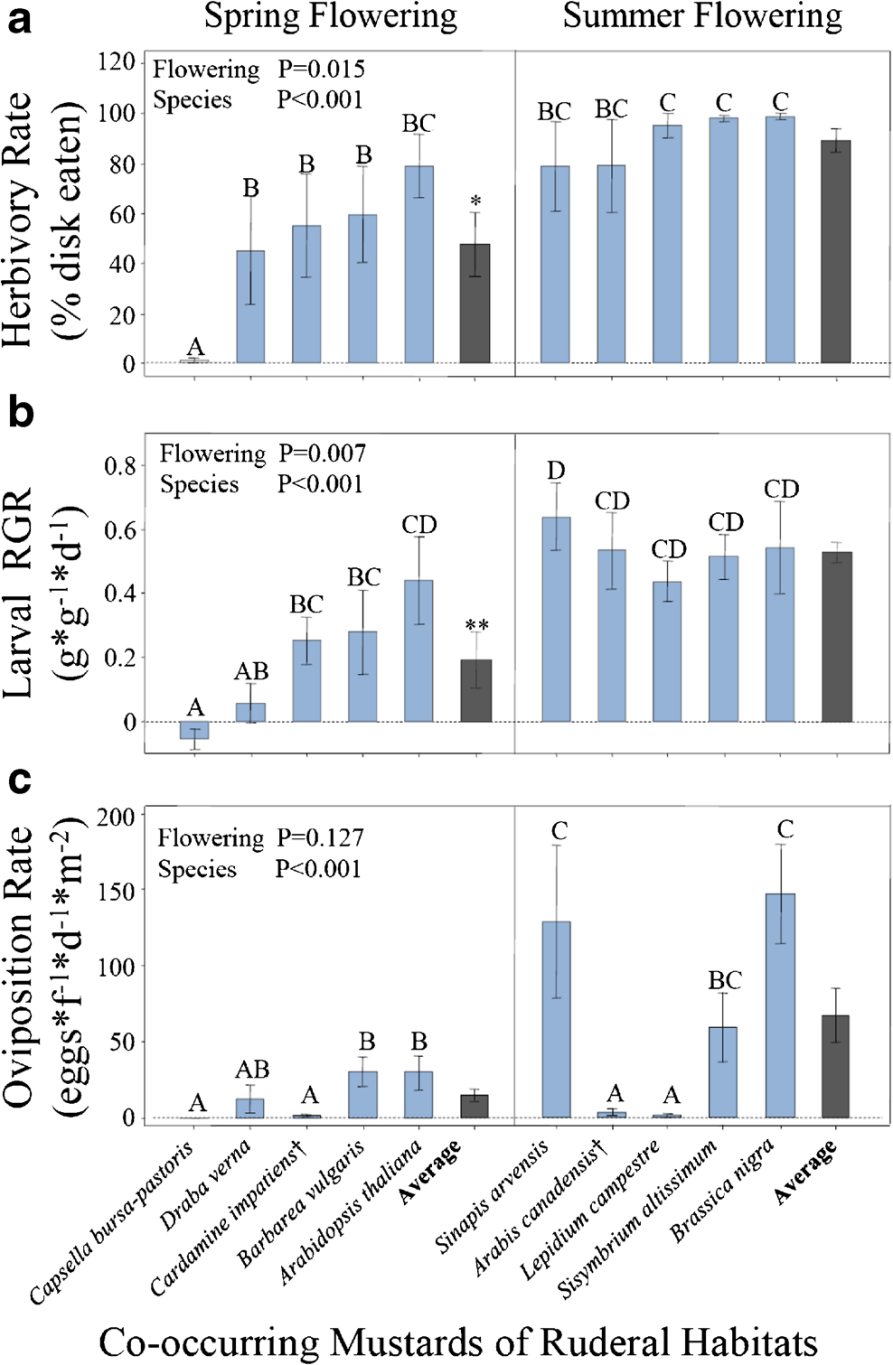
Comparison of **a**) herbivory (%) and **b**) relative growth rate (g*g^−1^*d^−1^) of *Pieris rapae* larvae in simultaneous disc feeding assays, and **c**) oviposition rates of females in choice arenas that included one individual plant of each of the ten species. Shown are means (+/− SE) for larval tests (*N* = 4 or 5) and adult female choice assays (*N* = 4). Significant differences at *P* = 0.05 between species are indicated by the absence of shared letters. Overall means (+/−SE) of the spring and summer groups are included (gray bars). P values are shown for flowering group and species nested within flowering group.*P < 0.05, **P < 0.01

When all host species were grown simultaneously under common garden conditions, the five spring-flowering mustards received significantly less herbivory (*F*_*1*, 8_ = 9.43, *P* = 0.015, Fig. 1a) and supported lower relative growth rates (*F*_*1*, 8_ = 13.24, *P* = 0.007, Fig. 1b) of first instars of *P. rapae* in the feeding assay relative to the five summer-flowering mustards (Table 1). The average leaf disk from the spring-flowering mustards lost 47.8 % of its area, whereas the average leaf disk from the summer-flowering mustards lost 89.9 % of its area, an amount nearly two-fold greater. The average larva feeding on a spring-flowering mustard disk gained 0.19 mg/mg of initial mass, whereas the average larva feeding on a summer-flowering mustard gained 0.53 mg/mg of initial mass over the same 24 h period, a roughly three-fold higher rate. Larvae were unable to consume *Capsella bursa-pastoris* and lost weight on those leaf disks. While this mustard was conspicuously resistant, it was not the only spring-flowering mustard that had high resistance to the larvae. *Draba verna*, *C. impatiens,* and *B. vulgaris* all exhibited substantially less damage and lower larval growth rates than the three most acceptable summer-flowering species. Of the spring-flowering species, only *A. thaliana* did not differ in quality relative to the summer-flowering species.

When gravid *P. rapae* females were offered an array including all ten species, the summer-flowering mustards received an average of 68 eggs/female/day/m^2^ of foliage, whereas the spring-flowering mustards received an average of less than 15 eggs/female/day/m^2^, which amounted to a four-fold difference, but this, however, was not statistically significant (*F*_*1*, 8_ = 2.89, *P* = 0.127, Fig. 1c), owing to strong differences among species. Two summer-flowering (*A. canadensis* and *L. campestre*) and two spring-flowering mustards (*C. bursa-pastoris* and *C. impatiens*) received essentially no eggs. If those four non-accepted species were removed, the remaining three summer-flowering mustards (*B. nigra, S. arvense*, and *S. altissimum*) had an average of 111 eggs/female/day/m^2^ of foliage, which was significantly greater than the 24 eggs/female/day/m^2^ received by the three spring-flowering mustards (*F*_*1*, 4_ = 10.1, *P* = 0.034).

**Fig. 2:**
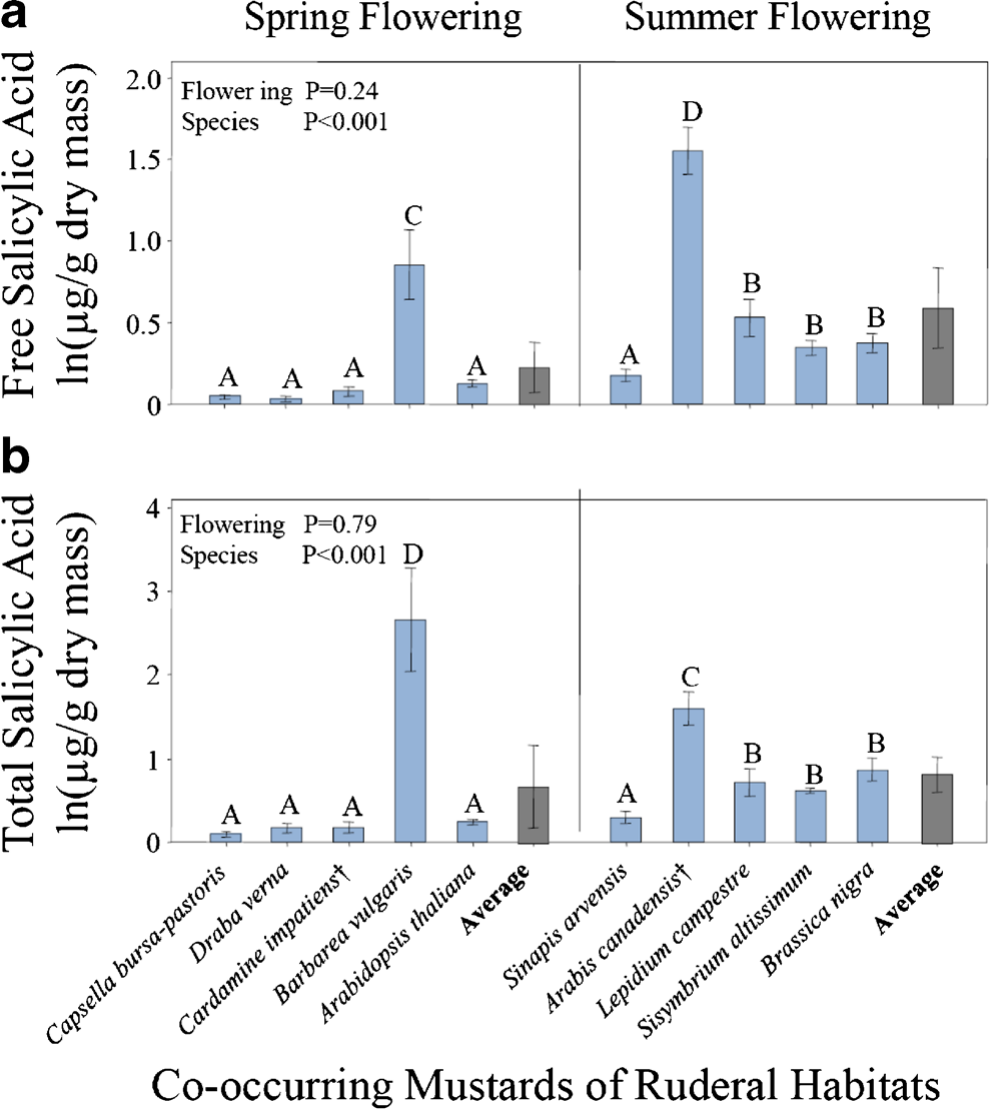
Comparison of natural log transformed values of constitutive **a**) free salicylic acid (SA, ug/g dry mass) and **b**) total SA (ug/g dry mass) of ten ruderal mustards. Shown are means (+/− SE) for leaf samples from four replicate plants. Significant differences at *P* = 0.05 between species are indicated by the absence of shared letters. Overall means (+/−SE) of the spring and summer groups are included (*gray bars*). *P* values are shown for flowering group and species nested within flowering group

Leaf constitutive SA concentrations did not differ significantly between the spring-flowering and summer-flowering species for either free SA (*F*_*1*, 8_ = 1.59, *P* = 0.242, Fig. 2a) or total SA (*F*_*1*, 8_ = 0.07, *P* = 0.793, Fig. 2b), when all species were included. However, one spring-flowering mustard (*B. vulgaris*) and one summer flowering mustard (*A. canadensis*) had unusually high concentrations of SA. When these two high concentration species were removed, the average of the four remaining summer-flowering mustards was 0.35 ug/g free SA, whereas the average of the four remaining spring-flowering mustards was 0.07 ug/g free SA, a nearly five-fold difference, which was significant (*F*_*1*, 6_ = 14.27, *P* = 0.009). For total SA, the difference between the average of the reduced set of four summer-flowering mustards and four spring-flowering mustards was 3.5-fold, which also was significant (*F*_*1*, 6_ = 12.80, *P* = 0.012).

**Fig. 3:**
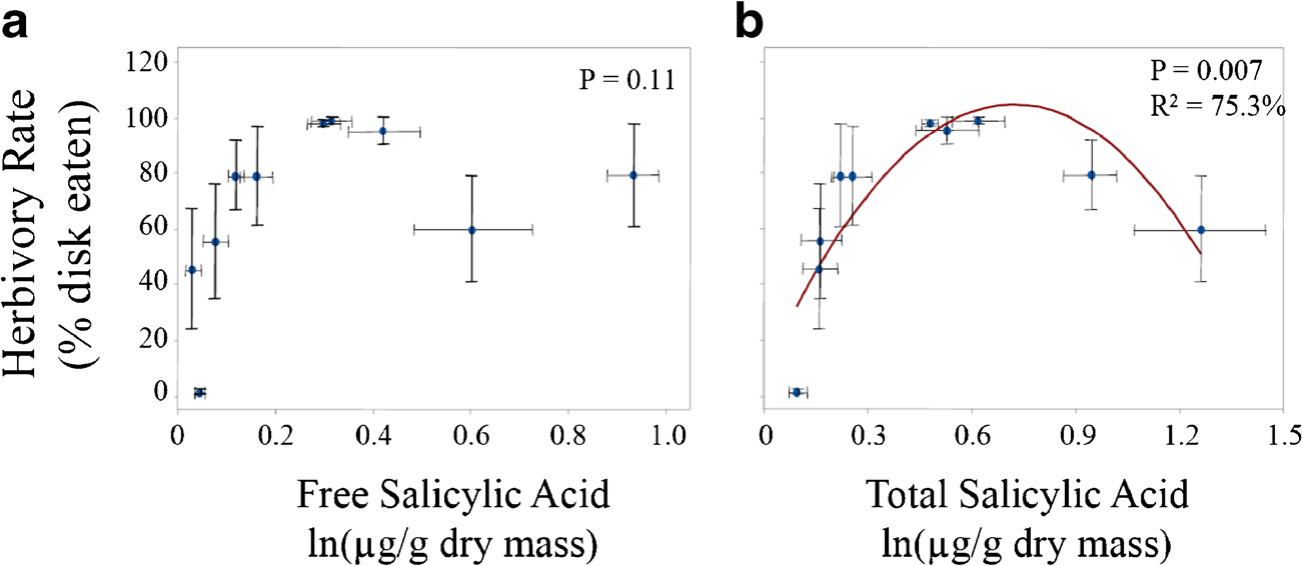
Scatterplots showing relationship between herbivory rate (% disk eaten) and constitutive leaf **a**) free salicylic acid (SA, ln(μg/g dry mass)) and **b**) total SA (ln(μg/g dry mass)) measured from plants reared in a separate experiment in the absence of herbivores. *P* values from polynomial regression are shown. *R* ^*2*^ value indicates percent variance in herbivory rate that is explained by the fitted polynomial regression line (Table 2). *Error bars* indicate +/− 1SE

**Fig. 4:**
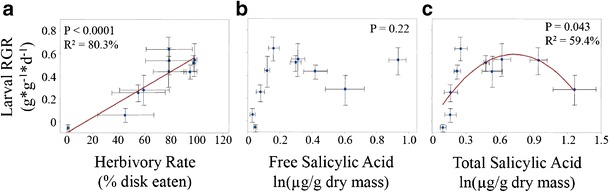
Scatterplots showing relationship between larval relative growth rate (RGR, g*g^−1^*d^−1^) and **a**) larval herbivory rate (% disk eaten), **b**) free salicylic acid (SA, ln(μg/g dry mass)) and **C**) total SA (ln(μg/g dry mass)) measured from plants reared in a separate experiment in the absence of herbivores. *P* values from linear or polynomial regression are shown. *R* ^*2*^ value indicates percent variance in larval relative growth rate that is explained by the fitted regression line (Table 2). *Error bars* indicate +/− 1SE

**Fig. 5:**
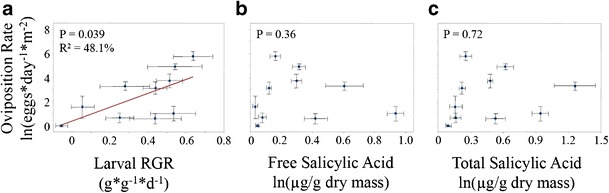
Scatterplots showing relationship between adult female oviposition rate (eggs*d^−1^*m^−2^) and **a**) larval relative growth rate (RGR, g*g^−1^*d^−1^), **b**) free salicylic acid (SA, ln(μg/g dry mass)) and **c**) total SA (ln(μg/g dry mass)) measured from plants reared in a separate experiment in the absence of herbivores. *P* values from linear or polynomial regression are shown. *R* ^*2*^ value indicates percent variance in larval relative growth rate that is explained by the fitted regression line (Table 2). *Error bars* indicate +/− 1SE

Leaf herbivory by *P. rapae* neonates was not correlated significantly with leaf constitutive free SA concentration (*R*^*2*^ = 46.5 %, *P* = 0.112, Fig. 3a, Table 2), but exhibited a strong polynomial relationship with total constitutive SA concentration (*R*^*2*^ = 75.3 %, *P* = 0.007, Fig. 3b, Table 2). Relative growth rate of these neonate larvae was correlated positively with the amount of the leaf disks consumed (*R*^*2*^ *=* 80.3 %, *P* < 0.001, Fig. 4a). Relative growth rate of the larvae was not correlated with leaf constitutive free SA concentration (*R*^*2*^ = 34.5 %, *P* = 0.227, Fig. 4b, Table 2), but exhibited a significant polynomial relationship with total constitutive SA concentration (*R*^*2*^ = 59.4 %, *P* = 0.043, Fig. 4c, Table 2). Female adult butterflies laid significantly more eggs on hosts that resulted in higher larval relative growth rates, as shown by the positive correlation between these two variables at the species level (*R*^*2*^ = 48.1 %, *P* = 0.039, Fig. 5a). Oviposition rates were not correlated with either the leaf constitutive concentrations of either free SA (*R*^*2*^ = 25.1 %, *P* = 0.363, Fig. 5b) or total SA (*R*^*2*^ = 9.0 %, *P* = 0.720, Fig. 5c).

**Table 2:** Regression models for larval % herbivory and performance as a function of leaf SA concentration

Response	Predictor	Source	DF	SS	F	P	R-Sq
% Herbivory	Ln(Free SA)	Full model	2	3800.72	3.0	0.112	46.5 %
		Linear	1	1257.55	1.5	0.262	
		Quadratic	1	2543.17	4.1	0.084	
		Error	7	4379.45			
		Total	9	8180.17			
	Ln(Total SA)	Full model	2	6160.76	10.7	0.007	75.3 %
		Linear	1	993.24	1.1	0.324	
		Quadratic	1	5167.52	17.9	0.004	
		Error	7	2019.41			
		Total	9	8180.17			
Larval RGR	Ln(Free SA)	Full model	2	0.15	1.8	0.227	34.5 %
		Linear	1	0.08	1.8	0.207	
		Quadratic	1	0.07	1.6	0.240	
		Error	7	0.30			
		Total	9	0.46			
	Ln(Total SA)	Full model	2	0.27	5.1	0.043	59.4 %
		Linear	1	0.05	1.0	0.353	
		Quadratic	1	0.22	8.4	0.23	
		Error	7	0.19			
		Total	9	0.46

**Fig. 6:**
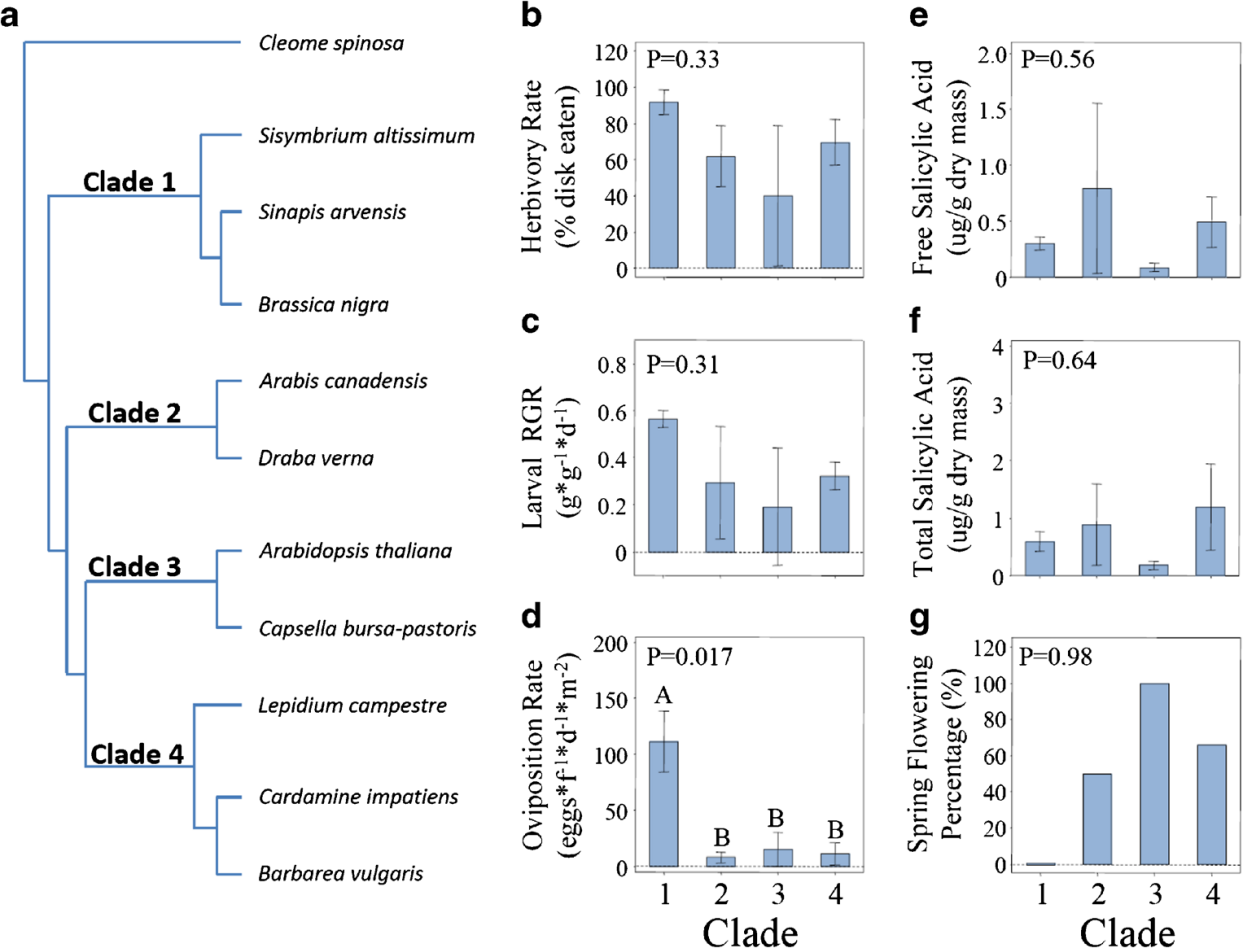
Assessment of the phylogenetic relationships among the mustards on their correlations with the performance of *Pieris rapae* and percentage of spring flowering phenology. **a**) Identification of four clades based on analysis of the maturase K (*matK*) gene sequences, with *Cleome spinosa* as the outgroup (Koch et al. 2001). Relationship between the four clades and average **b**) herbivory rates (% disk eaten), **c**) larval relative growth rate (g*g^−1^*d^−1^), **d**) oviposition rate (eggs*f^−1^*d^−1^*m^−2^), **e**) leaf free salicylic acid (SA, μg/g dry mass), **f**) leaf total SA (μg/g dry mass), and **g**) percentage of spring-flowering species in each group. *Error bars* indicate +/− 1SE

Phylogenetic grouping of the ten species based on maturase K (matK) gene sequences (Koch et al. 2001) resulted in the identification of four fully resolved clades, each containing two or three species (Fig. 6a). These clades explained significant variation in the oviposition rate by *P. rapae*, with Clade 1 (*S. altissimum*, *B. nigra,* and *S. arvense*) receiving seven-fold more eggs on average than species in the other three clades (*R*^*2*^ = 79.7 %, *P* = 0.017, Fig. 6d). These three species also all share the summer-flowering habit (Fig. 6f). Phylogenetic groupings did not explain significant variation in either herbivory rate (*R*^*2*^ = 41.0 %, *P* = 0.33, Fig. 6b), larval growth rate (*R*^*2*^ = 42.2 %, *P* = 0.31, Fig. 6c), leaf constitutive free SA concentration (*R*^*2*^ = 27.2 %, *P* = 0.56, Fig. 6e), or leaf constitutive total SA concentration (*R*^*2*^ = 22.7 %, *P* = 0.64, Fig. 6f).

## Discussion

In this study we asked whether differences in constitutive SA concentration among plants could predict larval performance of a specialist insect herbivore. To address this question, we measured SA in the leaves of ten mustard species and also determined relative growth rates and tissue damage by first-instar *P. rapae* caterpillars. We found that total constitutive SA concentration was indeed correlated with both *P. rapae* larval relative growth rate and the susceptibility of mustard plants to herbivory by first instar caterpillars. These results indicate that low SA concentration in this group of plants is correlated with high resistance to herbivory. While low SA concentrations have been linked previously to increased plant growth in the absence of enemies (Todesco et al. 2010), our data suggest that another possible benefit of low SA is elevated resistance to insect herbivores. Previous studies have shown that exogenous application of SA or analogs of SA directly increase plant susceptibility to insect herbivores (Cipollini et al. 2004; Thaler et al. 2002a, b, 2012), but this is the first study to show that constitutive levels of SA also are correlated with insect performance. Our data suggest that the relationship between constitutive SA concentration and resistance to *P. rapae* feeding is non-linear, with the highest resistance occurring at the lowest levels of SA. We see three possible mechanisms by which low constitutive levels of SA correlate with high plant resistance to insect herbivory.

First, this pattern could result from reduced negative pathway crosstalk between SA- and JA-mediated defenses. Here, low constitutive SA levels in leaves would have lower disruptive effects on constitutive or induced expression of defenses aimed at deterring herbivory, which are mediated by the hormone JA and suppressed by SA (Cipollini et al. 2004; Thaler et al. 2012; Traw and Bergelson 2003). Thus, low SA levels may allow for increased expression of JA-mediated defenses such as proteinase inhibitors and glucosinolates (Agrawal and Kurashige 2003), but may make a plant more susceptible to pathogen infection and disease development (Todesco et al. 2010). Measurement of JA concentrations or JA pathway expression would likely clarify the extent to which negative crosstalk explains the observed patterns. The correlation of higher total SA concentrations with susceptibility to herbivory suggests an ecological cost of maintaining a high level of free SA in plant tissues.

Second, it also is possible that SA itself directly stimulated insect feeding and performance (van Loon 1990; but see Akbar et al. 2012; Raju et al. 2009). The extent to which herbivores can perceive SA is unknown. However, a molluscan herbivore has been shown recently to secrete SA onto plant tissues (Kastner et al. 2014) and a whitefly has been shown to convert free SA into its glycoside and secrete this glycoside onto leaves (VanDoorn et al. 2015). Finally, both SA levels and resistance to insect feeding may not have a causal relationship, but instead be both correlated with a third factor, such as flowering time and / or leaf nitrogen concentration. Salicylic acid promotes flowering in *Arabidopsis thaliana* (Jin et al. 2008; Martinez et al. 2004) and other species (Wada et al. 2010). Salicylic acid concentrations are higher in the leaves of flowering tobacco plants (Yalpani et al. 1993). Nitrogen is known to be limiting to growth rates of *P. rapae* (Slansky and Feeny 1977), but whether leaf nitrogen and SA contents are correlated remains unknown. For these reasons, manipulative experiments will be required to distinguish a causal effect of SA on insect performance from other possibly correlated variables.

Total SA concentrations include the large fraction of SA that is conjugated to sugar (Vlot et al. 2009). We found that total constitutive SA had a stronger correlation with insect herbivory and performance than did free SA alone. Surprisingly, one of the mustard species, *Arabis canadensis*, produced only free SA and did not have any sugar-conjugated SA (Supplemental Fig 1). In the absence of this outlier, both tissue consumption and relative growth rates were correlated with leaf constitutive free SA concentration (Supplemental Fig 2, Supplemental Table 1). Collectively, our data suggest that both constitutive levels of total and free SA are generally tightly correlated with each other and herbivore performance. Therefore, it is not possible to distinguish between the relative ecological importance of the free and sugar-conjugated forms at present.

Our data suggest that ovipositing females of *P. rapae* were quite conservative in their behavior, laying eggs on fewer species than would support larval growth, but none on the lethal and near-lethal options. Thus, essentially all eggs were laid on suitable hosts. However, *P. rapae* did not lay eggs on two species that were superior hosts for larval growth, *Arabis canadensis* and *Lepidium campestre*, and one host that allowed moderate larval growth, *Cardamine impatiens*. Thus, they excluded three of the eight hosts that could support larval growth. However, they correctly excluded *Capsella bursa-pastoris* and *Draba verna*, which were unsuitable for larval growth, as has been shown previously (Renwick and Radke 1987). For female *P. rapae* butterflies in the wild, this conservative strategy would most probably result in an increased time spent locating hosts, but lower neonate mortality, relative to a strategy that utilized all of these mustards equally. Our results provide a strong degree of correspondence with previous work on the closely related butterfly, *Pieris napi* (Chew 1977). In its habitat in the Rocky Mountains, *P. napi* typically lays eggs on mustard hosts that provide the strongest larval performance, while also avoiding the local *Draba* and *Lepidium* species.

Interestingly, adult females in our study did appear to have a preference for the exclusively summer-flowering Clade 1 (Fig. 6d). Whether this association reflects convergent properties of the hosts to this environment or phylogenetic association with this particular clade of mustards (Braby and Trueman 2006; Ehrlich and Raven 1964; Janz 2011) remains unknown.

The current study does not address intraspecific variation, which other studies have shown can be substantial with respect to leaf SA concentration (Silverman et al. 1995; Zhang et al. 2014) and the performance of *Pieris* larvae (Chew 1975). Broad conclusions about the host suitability and relative leaf SA contents of these ten mustards are, therefore, not warranted. An important next step will be the measurement of leaf SA concentrations for a broader sample of genotypes and under a greater range of environmental conditions.

*Pieris rapae* butterflies have between three and seven generations each year (Gaines and Kok 1995; Maltais et al. 1998). The exact number of generations, development time, and earliest spring emergence are all dependent on local environmental factors, especially average ambient temperature. Our finding that spring-flowering mustards in this study tend to have lower SA levels and greater resistance to herbivory by *P. rapae* may reflect pressure from other environmental factors such as infection by pathogens or competition from neighboring plants. The impact of these environmental factors on plant defense chemistry is an area where more research is needed. *Barbarea vulgaris* often is the first host used by overwintering *Pieris* females in the spring for oviposition (Gaines and Kok 1995). Interestingly, our data suggest that this is a weak preference. When the females had the option of the summer mustards under common garden conditions, they preferred most of them over *B. vulgaris.*

*Pieris rapae* caterpillars feed widely on plants in the family Brassicaceae including a number of crop species, and the caterpillars can be an important agricultural pest (Capinera 2001; Lasota and Kok 1989). Previous work has found that *P. rapae* caterpillar numbers are higher later in the growing season (Gaines and Kok 1995; Maltais et al. 1998) and that early-flowering cultivars of broccoli are hosts to lower numbers of caterpillars of various species when compared to later-flowering cultivars (Vail et al. 1991). Our work suggests a physiological explanation for this observation. If these cultivars have lower constitutive SA levels, it may preclude the inhibition of JA-dependent defenses via negative crosstalk. With higher JA-dependent defenses, these plants would then support lower numbers of insects than summer-flowering cultivars with higher SA concentrations.

In summary, the current study presents some of the first evidence that constitutive concentrations of SA in host plants may influence insect herbivory and performance. Experimental manipulation of SA levels in these species will be an important goal of future work. Because SA is found almost universally in plants and can be readily assayed, it may have potential as a common quantitative predictor of basal resistance. Further understanding of this relationship may influence crop breeding and selection for resistance to insect herbivores.

## Electronic supplementary material

Supplemental Table 1 (DOC 108 kb)

Supplemental Fig 1 (DOC 138 kb)

Supplemental Fig 2 (DOC 157 kb)
