# Supplementary material for: Herbivory and Relative Growth Rates of Pieris rapae are Correlated with Host Constitutive Salicylic Acid and Flowering Time
Source: J Chem Ecol. 2015 Apr 17;41(4):350–9. doi: 10.1007/s10886-015-0572-z (PMC4427633; doi:10.1007/s10886-015-0572-z)
Supplement: Supplementary file 1 — (DOC 108 kb) [file 10886_2015_572_MOESM1_ESM.doc]

**Table S1.** Polynomial regressions for % herbivory and larval relative growth rate as a function of natural log transformed constitutive free salicylic acid concentration excluding *Arabis canadensis*.
