# Supplementary material for: Herbivory and Relative Growth Rates of Pieris rapae are Correlated with Host Constitutive Salicylic Acid and Flowering Time
Source: J Chem Ecol. 2015 Apr 17;41(4):350–9. doi: 10.1007/s10886-015-0572-z (PMC4427633; doi:10.1007/s10886-015-0572-z)
Supplement: Supplementary file 2 — (DOC 138 kb) [file 10886_2015_572_MOESM2_ESM.doc]

**Fig. S1.**  Relationship between total and free leaf constitutive salicylic acid concentrations for all ten species of mustard studied. *Arabis canadensis* (Ac) is an exception in that it falls on the y = x line, suggesting that none of its salicylic acid was conjugated to sugar.
