# Supplementary material for: Herbivory and Relative Growth Rates of Pieris rapae are Correlated with Host Constitutive Salicylic Acid and Flowering Time
Source: J Chem Ecol. 2015 Apr 17;41(4):350–9. doi: 10.1007/s10886-015-0572-z (PMC4427633; doi:10.1007/s10886-015-0572-z)
Supplement: Supplementary file 3 — (DOC 157 kb) [file 10886_2015_572_MOESM3_ESM.doc]

**Fig. S2.** Scatterplots showing relationship between A) herbivory rate (% disk eaten) and B) larval relative growth rate as a function of leaf constitutive free salicylic acid ln(μg/g dry mass) excluding *Arabis canadensis*  (Ac). *P* values from polynomial regression are shown. *R^2^* value indicates percent variance in herbivory rate that is explained by the fitted polynomial regression line. Error bars indicate +/- 1SE.
